# Comment: In Response to “Downgraded Lymphoma: B-Chronic Lymphocytic Leukemia in a Known Case of Diffuse Large B-Cell Lymphoma - De Novo Occurrence or Transformation”

**DOI:** 10.4274/tjh.2015.0452

**Published:** 2016-05-16

**Authors:** Burak Uz, Kadir Acar

**Affiliations:** 1 Gazi University Faculty of Medicine, Department of Internal Medicine, Division of Adult Hematology, Ankara, Turkey

**Keywords:** Diffuse large B-cell lymphoma, Downgraded lymphoma

## TO THE EDITOR

We read the letter submitted by Gajendra et al. with deep interest [1]. The authors described a patient diagnosed with diffuse large B-cell lymphoma (DLBCL) non-germinal center B-cell type in 2002 who received 6 cycles of cyclophosphamide, adriamycin, vincristine, and prednisolone (CHOP) followed by radiotherapy. He was well for nearly 5 years, but subsequently his disease locally relapsed. Unfortunately, a planned intensive salvage regimen could not be given because the patient was lost to follow-up. In 2010, despite not being given any treatment modality, he presented with small lymphocytic lymphoma. Finally, 22 months thereafter, he was diagnosed with Rai stage IV chronic lymphocytic leukemia and 6 cycles of fludarabine, cyclophosphamide, and rituximab (FCR) were administered, resulting in complete remission.

As is known, indolent or low-tumor-burden lymphomas may transform into aggressive or high-tumor-burden lymphoma forms in a process called “Richter transformation”. Although rare, the reverse process may also occur with unknown mechanisms. At this point, there are two main hypotheses that can be suggested: initially, there are two existing malignant clones, and successful eradication of the rapidly proliferating clone with intensive therapy results in the survival of the less rapidly growing clone, which may eventually lead to relapsed disease even many years following the diagnosis [2]; or, less probable, a separate secondary malignant clone that is distinct from the initial clone might appear [3].

Previously, two downgraded lymphoma cases were reported [2,3] after the successful treatment of underlying diffuse non-Hodgkin lymphoma, 3 and 14 years following the initial diagnosis. This well-described patient was accepted as having late-relapsed (~5 years later) DLBCL, which transformed into a “downgraded lymphoma” without lymphoma-specific therapy. DLBCL patients generally relapse in the first 2 or 3 years following treatment. The largest series of patients with DLBCL who relapsed ≥5 years following diagnosis was reported by a French group [4]; 3.6% of their cohort had a late relapse and those patients had some distinct clinical features, including localized disease (63%), favorable International Prognostic Index score (82%), and extranodal involvement (65%) at diagnosis. At the time of relapse, 83% had DLBCL histology, while 17% had indolent histology. Additionally, having an indolent component at diagnosis (44.4%) was significantly associated with indolent histology at relapse. However, nearly all the late-relapsed patients with initial good-risk disease were treated adequately with anthracycline-based combined chemotherapy.

Late-relapsed DLBCL patients have poor outcomes; therefore, they must be treated promptly with rituximab plus chemotherapy or (if possible) autologous stem cell transplantation [4]. In the French experience, all late-relapsed patients were heavily treated and the patients experienced their relapse a median of 7.4 years after diagnosis [4]. As an interesting aside, the present patient could not be administered any treatment for 3 years after the confirmation of DLBCL relapse and he presented with downgraded lymphoma. We could not understand why the patient’s relapsed high-grade lymphoma resolved without any treatment attempts. Spontaneous remission of DLBCL is exceedingly rare, with only a handful of such cases reported [5,6,7,8]. Given the unexplained clinical course of DLBCL in this patient, a probable infectious agent or nonprescription usage of traditional medicinal plants inducing antitumor response by modulating the immune system against lymphomatous cells should be sought in his medical history.
